# Quantitative and Qualitative Responses to Topical Cold in Healthy Caucasians Show Variance between Individuals but High Test-Retest Reliability

**DOI:** 10.1371/journal.pone.0151972

**Published:** 2016-03-23

**Authors:** Penny Moss, Jasmine Whitnell, Anthony Wright

**Affiliations:** School of Physiotherapy and Exercise Science, Curtin University of Technology, Perth, Western Australia; University of Ottawa, CANADA

## Abstract

Increased sensitivity to cold may be a predictor of persistent pain, but cold pain threshold is often viewed as unreliable. This study aimed to determine the within-subject reliability and between-subject variance of cold response, measured comprehensively as cold pain threshold plus pain intensity and sensation quality at threshold. A test-retest design was used over three sessions, one day apart. Response to cold was assessed at four sites (thenar eminence, volar forearm, tibialis anterior, plantar foot). Cold pain threshold was measured using a Medoc thermode and standard method of limits. Intensity of pain at threshold was rated using a 10cm visual analogue scale. Quality of sensation at threshold was quantified with indices calculated from subjects' selection of descriptors from a standard McGill Pain Questionnaire. Within-subject reliability for each measure was calculated with intra-class correlation coefficients and between-subject variance was evaluated as group coefficient of variation percentage (CV%). Gender and site comparisons were also made. Forty-five healthy adults participated: 20 male, 25 female; mean age 29 (range 18–56) years. All measures at all four test sites showed high within-subject reliability: cold pain thresholds r = 0.92–0.95; pain rating r = 0.93–0.97; McGill pain quality indices r = 0.87–0.85. In contrast, all measures showed wide between-subject variance (CV% between 51.4% and 92.5%). Upper limb sites were consistently more sensitive than lower limb sites, but equally reliable. Females showed elevated cold pain thresholds, although similar pain intensity and quality to males. Females were also more reliable and showed lower variance for all measures. Thus, although there was clear population variation, response to cold for healthy individuals was found to be highly reliable, whether measured as pain threshold, pain intensity or sensation quality. A comprehensive approach to cold response testing therefore may add validity and improve acceptance of this potentially important pain measure.

## Introduction

Increased sensitivity to cold may be a robust predictor of persistent pain [1,2,3] or of increased post-operative pain [4]. Cold hyperalgesia has been reported as an important characteristic of neuropathic pain [5,6,7] and also in some individuals with less clearly neuropathic disorders such as fibromyalgia [8,9] whiplash associated disorder, [10,3], spinal pain [11,12] and osteoarthritis [13,14].

Despite this increasingly use of cold response data, literature reporting the reliability of cold pain measures is limited. A recent systematic review of quantitative sensory testing (QST) concluded that, in contrast to other QST measures, cold pain threshold (CPT) reliability is not yet well established, largely due to limited published data [15]. This lack of data may be partly explained by the floor effect from the 5°C cut-off temperature of thermodes used by many studies, which means that significant numbers of healthy participants do not reach cold pain threshold, resulting in exclusion of CPT from analysis [16].

The limited available CPT reliability data is ambiguous, complicated by considerable methodological variability between studies, particularly as regards thermode type, test sites and statistical analysis. For example, Heldestad et al. [17] investigated variation in volar forearm CPT in healthy subjects, reporting very low intra-subject coefficients of variation (CV) of 0.73%, 1.6% and 0.63%. However, investigators were required to use a 10°C cut off temperature, thus artificially limiting variability. Geber et al. [18] used the TSA II thermode (Medoc, Israel) which has a cut-off temperature of 0°C and reported within-subject test-retest Intra-Class Correlation Coefficients (ICC) for CPT of between r = 0.86 and r = 0.79 across non-standardised test sites in 60 participants. Moloney at al. [19] also used the Medoc thermode and reported intra-subject ICCs of between r = 0.87 and r = 0.94 for CPT at the second digit. However, this study also reported very high variability between individuals with between-subject CVs of between 84.9% and 90.2%. There is clearly a need for additional CPT reliability data, which clearly delineates within-subject and between-subject results.

Additional sensory evaluation of cold response, such as intensity or quality of sensation experienced during cold stimulus would offer a more comprehensive assessment of cold sensitivity. However, only a very few studied have included any such additional sensory measures, with only two considering test-retest reliability. Wasner and Brock [20] asked subjects to quantify intensity of pain and cold or heat during CPT on numerical rating scales and found high test-retest reliability in both (ICCs between r = 0.80 and .r = 0.94). Geber et al. [18] recorded number of paradoxical heat sensations experienced during cold stimuli, reporting a less strong correlation between two test sessions (r = 0.351). However, no previous study has analysed the test-retest reliability of both pain intensity and a comprehensive assessment of sensation quality at cold pain threshold.

The current study therefore aimed to determine more comprehensively the within-subject test-retest reliability and between-subject variability of sensory response to cold in healthy adults over three separate test sessions at two upper limb and two lower limb sites. Response was measured using conventional CPT temperature and pain intensity sensation quality ratings at CPT as well as cold detection thresholds (CDT).

## Methods

### Study Design

A repeated measures design was used, with response to cold (CPT, pain intensity at CPT and pain quality at CPT) tested at four body sites on three separate occasions. Cold detection thresholds were also tested on each occasion and at each site. Using data from a previous study [13] it was calculated that a sample size of between 40 and 50 subjects would provide 80% power (α = 0.05).

### Participants

Subjects were recruited voluntarily, via word-of-mouth and advertisements, from the Perth W.A. metropolitan area. All subjects were adults over 18 years of age, with normal sensation at the test sites and able to read and understand written and spoken English. Only volunteers of Caucasian ethnicity were recruited in order to limit ethnic variability in cutaneous pain thresholds [21,22]. Exclusion criteria included the presence of any acute pain, history of any chronic pain condition or neurological disorder and intake of any pain medication within 24 hours of testing. All volunteers provided written informed consent prior to participating. Approval was obtained from the Curtin University Human Research Ethics Committee (Approval Number PT0188).

### Procedure

**Fig 1:**
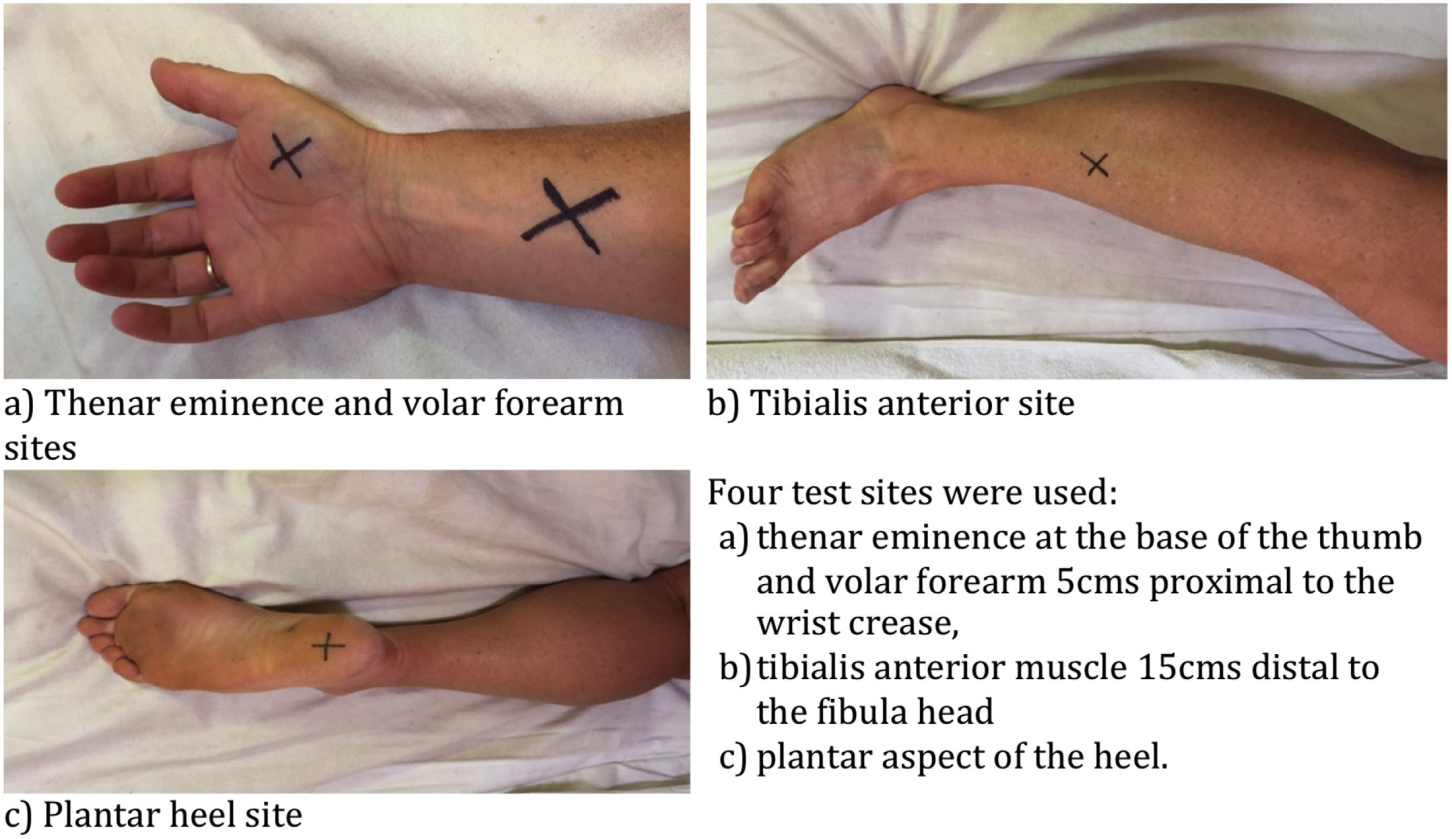
Test sites.

All subjects attended three test sessions, each separated by at least 24 hours. All testing was performed by the same investigator and standardized, scripted instructions given for each task. Four sites on the non-dominant side were tested in randomized order (Fig 1).

### Outcome Measures

#### Cold detection and cold pain thresholds

Cold detection threshold (CDT) was tested first at each site. Any subject who failed to detect cold within 10°C of the starting temperature was excluded from further testing. Cold pain threshold (CPT) tests were then completed at each site. All assessment was performed with a Thermal NeuroSensory Analyzer (TSA) 2001-II (MEDOC, Israel) and stimulus delivery controlled by TSA software. A thermode probe with a contact area of 15cm^2^ was securely strapped to the test site. All CDT and CPT testing followed the standard Method of Limits protocol [23]. The temperature decreased at a rate of 1°C/s from a baseline of 32°C to a cut-off of 0°C. For CDT, subjects were instructed: “Please press your hand-held switch as soon as you sense any cold sensation”. For CPT subjects were instructed: “Please press your switch as soon as the sensation of cold changes to one of uncomfortable or painful cold”. Once the subject pressed the control switch, or once 0°C was reached, the temperature returned to baseline. For each site and each threshold type, a practice was followed by three trials, each separated by a variable pause of between 4 and 6 seconds. The mean value was used for analysis. For subjects who did not press the control switch before the cut-off temperature, CPT value was defined as 0°C.

#### Pain intensity of sensation at CPT

On completion of CPT testing at each site subjects were asked to rate the intensity of pain experienced at CPT, using a 100mm visual analogue scale (VAS). Visual analogue scales have demonstrated concurrent validity with other pain measurement methods [24] and good reliability [25].

#### Quality of sensation at CPT

Participants were also asked to select words from the McGill Short-Form Questionnaire (SF-MPQ) (Part 2) [26] to describe sensation quality at CPT. This was completed once per test session. The SF-MPQ (part 2) includes a list of 70 sensory, affective and general descriptors. Two indices can be calculated: the Pain Rating Index (PRI) is the sum of the values allocated to each word [27] the number of words counted (NWC) is the total number of words chosen [26]. The SF-MPQ has been deemed valid and reliable (*r =* .96) [28] and has previously been used in experimental pain studies with healthy subjects [29].

### Sample Size and Data Analysis

Data were analyzed using SPSS for Mackintosh v22 (IBM Corp., NY), with alpha set at *p*≤0.05. Shapiro-Wilk analysis showed that only CPT values at the thenar eminence and foot were normally distributed. All non-normal data was log transformed for further analysis. Within-subject reliability across the three test sessions was calculated for each test site using Intra-Class Correlation Coefficients (ICC) (2-way absolute model) for mean CPT, VAS pain and McGill indices at CPT and for mean CDT. Within-days variance for each measure at each site was expressed as Coefficient of Variation percentage (CV%), using the standard deviation (SD) of between-days means divided by the between-days mean x100. Between-subject variance was explored through calculation of group means, standard deviations and inter-quartile ranges for each site at each test session. Group CV% was also calculated for each test session at each site ((group SD/group mean)x100). Skewness and kurtosis values were also calculated for each measure at each site on each test occasion to further explore data distribution.

Influence of site and gender were also analysed. Differences between sites were analysed using either Repeated Measures ANOVA or Friedman’s 2-way Analysis of Variance (session and site, or session and gender as factors). Independent t-tests / Mann-Whitney U test evaluated whether there was a gender difference in CPT, VAS and McGill scores. Gender-separated ICCs, within-days CV% and group CV% were calculated to evaluate differences in within-subject test-retest reliability and between-subjects variance between genders.

## Results

Forty-five participants completed all three testing sessions: 20 male and 25 female; mean age 29 (range 18–56) years. There were no significant differences in age between genders (p = 0.466). No subjects were withdrawn from the study.

### Cold Pain Threshold

Within-subject reliability for CPT across the three test sessions was high at each test site. ICCs ranged from *r =* .92 to r = .95 (p < .001) (Table 1). Mean CPT was consistently higher at the two upper limb sites (mean 11.21°C) compared with the lower limb sites (mean 9.07°C). Between-days variance for CPT across the three test sessions ranged from CV% of 1.16% for the forearm to 9.08% for the foot.

**Table 1:** Within-subject test-retest reliability data.

		Test session mean values			Mean (SD) over 3 test sessions		ICC**	CV%
Site		1	2	3			r (95% CI)	
**CPT**	Thenar	10.78	11.65	10.73	11.08	(0.50)	0.93 (0.89–0.96)	4.48
**(°C)**	Forearm*	11.51	11.34	11.25	11.36	(0.13)	0.94 (0.90–0.97)	1.16
	Tibialis Ant.*	9.72	9.15	8.13	9.04	(0.74)	0.93 (0.88–0.97)	8.22
	Foot	10.04	8.99	8.38	9.14	(0.83)	0.94 (0.91–0.97)	9.08
**VAS**	Thenar	3.59	3.47	3.68	3.58	(0.11)	0.96 (0.94–0.98)	2.96
**(/10)**	Forearm*	3.66	3.40	3.23	3.43	(0.21)	0.93 (0.88–0.96)	6.20
	Tibialis Ant.*	2.54	2.60	2.56	2.57	(0.03)	0.95 (0.91–0.97)	1.15
	Foot	3.12	2.79	2.84	2.92	(0.18)	0.94 (0.90–0.97)	6.12
**McGill Descriptor Indices**								
	NWC*	4.73	4.44	4.44	4.53	(0.15)	0.89 (0.82–0.94)	3.32
	PRI*	10.73	10.44	10.38	10.51	(0.17)	0.86 (0.77–0.92)	1.62

CPT Cold Pain Threshold; VAS Visual Analogue Scale; NWC Number of Words Chosen; PRI Pain Rating Index SD Standard Deviation; ICC Intra-Class Correlation Coefficient; CI Confidence Interval; CV Coefficient of Variation.

* Log transformation required before ICC calculation, due to non-normal distribution;

** *p*<0.001 for all ICC values

Between-subjects variance for CPT was considerably larger, with group standard deviations and interquartile ranges consistently high relative to the population mean (Table 2).

**Table 2:** Between-subject means, standard deviations (SD), interquartile ranges and group coefficient of variation (CV%) for cold response measures.

		Test Session 1				Test Session 2				Test Session 3			
	Site	Mean	SD	IQ Range	Group CV%	Mean	SD	IQ Range	Group CV%	Mean	SD	IQ Range	Group CV%
**CPT**	Thenar	10.78	6.66	10.77	**61.8**	11.65	6.80	12.10	**58.4**	10.80	6.82	11.10	**63.2**
**(°C)**	Forearm	11.51	8.83	15.85	**76.7**	11.33	8.10	13.14	**71.5**	11.25	7.40	10.89	**65.8**
	Tib Ant	9.73	8.94	19.09	**91.9**	9.15	8.57	16.78	**93.7**	8.25	7.61	13.52	**92.2**
	Foot	10.04	6.57	7.48	**65.4**	8.99	5.46	6.14	**60.1**	8.40	5.23	6.37	**62.3**
**VAS**	Thenar	3.59	2.38	4.50	**66.3**	3.47	2.37	4.25	**68.3**	3.68	2.60	4.50	**70.7**
**(/10)**	Forearm	3.66	2.50	4.75	**68.3**	3.40	2.41	4.00	**70.9**	3.23	2.50	4.50	**77.4**
	Tib Ant	2.54	2.19	3.50	**86.2**	2.60	2.40	3.00	**92.3**	2.56	2.37	3.50	**92.5**
	Foot	3.12	2.09	3.50	**66.9**	2.79	2.14	3.50	**76.7**	2.84	2.29	4.00	**82.2**
**McGill Descriptor Indices**													
	NWC	4.73	2.43	3.00	**51.4**	4.44	2.10	3.00	**47.3**	4.44	2.28	2.50	**51.4**
	PRI	10.73	5.84	8.00	**54.4**	10.44	5.14	6.50	**49.2**	10.38	6.15	10.50	**59.2**

CPT Cold Pain Threshold; VAS Visual Analogue Scale; NWC Number of Words Chosen; PRI Pain Rating Index

### Pain intensity at CPT

Within-subjects reliability for VAS rating of pain intensity at CPT was also consistently high (ICCs r = 0.93 thenar to r = 0.97 tibialis anterior (Table 1). Between-days variance ranged from CV% of 1.15% at tibialis anterior to 6.20% at the forearm. VAS pain ratings at CPT were relatively low, averaging 3/10. The thenar eminence exhibited greatest pain at CPT (mean VAS 3.6 ± 0.1) with the least pain experienced at the tibialis anterior site (mean VAS 2.6 ± 0.03).

Between-subjects variance for pain intensity at CPT was similarly greater than within-subject variance (Table 2). At each site, VAS intensity ranged from 0/10 to 8.5/10. Group data were also positively skewed at all sites and kurtosis values indicated a generally flat data distribution (Table 3).

**Table 3:** Within-subject test-retest reliability, comparing males and females.

		Males (n = 20)					Females (n = 25)				
		Mean (SD) for 3 sessions		ICC**		CV%	Mean (SD) for 3 sessions		ICC**		CV%
	Site			r (95% CI)					r (95% CI)		
**CPT**	Thenar	8.86	(0.79)	0.90	(0.79–0.96)	8.89	12.85	(0.26)	0.94	(0.89–0.97)	2.05
**(°C)**	Forearm*	8.67	(0.38)	0.92	(0.83–0.97)	4.42	13.52	(0.44)	0.96	(0.92–0.98)	3.28
	Tibialis Ant.*	6.26	(0.76)	0.95	(0.90–0.98)	12.18	11.02	(0.70)	0.97	(0.94–0.99)	6.37
	Foot	6.74	(0.68)	0.90	(0.78–0.96)	10.04	11.06	(0.96)	0.94	(0.88–0.97)	8.66
**VAS**	Thenar	3.18	(0.13)	0.90	(0.79–0.96)	4.17	3.80	(0.29)	0.96	(0.92–0.98)	7.76
**(/10)**	Forearm*	3.10	(0.34)	0.92	(0.83–0.97)	10.91	3.72	(0.09)	0.94	(0.88–0.97)	2.55
	Tibialis Ant.*	1.95	(0.20)	0.95	(0.90–0.98)	10.26	3.14	(0.15)	0.92	(0.84–0.96)	4.80
	Foot	2.44	(0.27)	0.90	(0.78–0.96)	11.23	3.49	(0.12)	0.92	(0.85–0.96)	3.31
**McGill Descriptor Indices**											
	NWC*	4.18	0.37	0.66	0.30–0.86	8.81	4.83	0.06	0.95	0.91–0.98	1.27
	PRI*	9.40	1.08	0.70	0.38–0.87	11.45	11.41	0.55	0.93	0.86–0.97	4.78

CPT Cold Pain Threshold; VAS Visual Analogue Scale; NWC Number of Words Chosen; PRI Pain Rating Index SD Standard Deviation; ICC Intra-Class Correlation Coefficient; CI Confidence Interval; CV Coefficient of Variation.

* Log transformation required before ICC calculation, due to non-normal distribution;

** *p*<0.001 for all ICC values.

### Sensation quality at CPT

**Fig 2:**
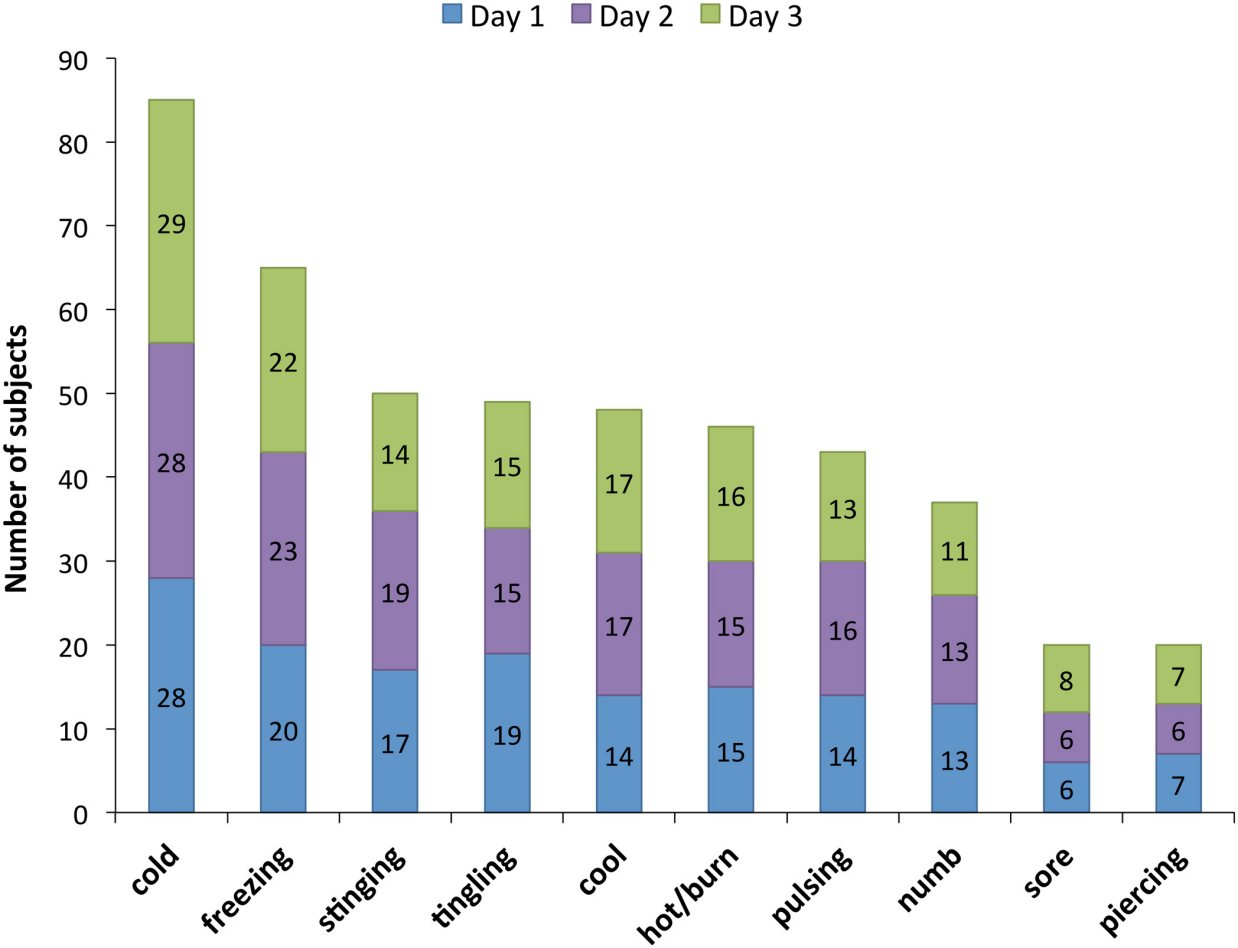
Sensation quality descriptors at each test session. There was high consistency of the most frequently chosen descriptors of sensation quality at cold pain threshold across the three test sessions.

Within-subject reliability for sensation quality was also high (ICC for NWC *r =* 0.87 (p < .001); ICC for PRI *r =* 0.85 (p < .001)) and between-days variance was low (CV% 2.51% for NWC and 1.31% for PRI) (Table 1). Fig 2 illustrates the consistency of word choice between test sessions. The basic sensation of cold was almost universally experienced at CPT. Paradoxical heat-type words, usually associated with an abnormal response, were chosen by 12 of these pain-free participants (27%), almost always in combination with a strong cold word such as "freezing".

As for CPT and VAS, between-subject variance for sensation quality was wide, with large group SDs and interquartile ranges for both descriptor index values (Table 2). Data for both indices were positively skewed with low kurtosis values (Table 3). Fig 1 illustrates the range of sensory qualities reported, each word selected by at least 3 participants. Although 93% of participants selected at least one cold-type word, most selected ‘cold’ or ‘freezing’. Paradoxical heat in combination with cold was reported by 15% of participants. Three subjects (7%) reported paradoxical burning with no cold.

### Cold detection threshold

Within-subject reliability for cold detection threshold was considerably lower than for cold pain threshold, with ICCs ranging from r = 0.75 for the foot to r = 0.56 for the forearm. In contrast to CPT, between-subjects variance was less, with smaller SDs and interquartile ranges relative to the mean. CDT distribution was negatively skewed and generally exhibited higher Kurtosis values than for CPT.

### Influence of test site location

Repeated Measures ANOVA was applied to investigate differences between test sites in CPT values. There were differences between sites on all test occasions, with upper limb sites consistently exhibiting higher CPT values than lower limb sites (Table 1). This difference reached statistical significance on Days 2 (p = 0.002) and 3 (p = 0.001) although not on Day 1 (p = 0.102). Pain ratings at CPT also varied between test sites, with upper limb sites consistently evoking significantly higher pain ratings than lower limb sites (combined upper limb means: 3.63, 3.44 and 3.46 /10; combined lower limb means: 2.83, 2.70, 2.70 / 10). Investigation of CDT values showed that the foot was consistently less sensitive than the other test sites on all test occasions (all p<0.001).

### Influence of gender

CPT values were significantly higher for females than for males, at all but the tibialis anterior site, although there was no significant gender difference in pain rating at CPT at any site (p = 0.270 to p = 0.435), or in sensation quality (NWC p = 0.747; PRI p = 0.814).

Female subjects showed greater reliability for CPT testing at all sites (ICCs r = 0.94–0.97) compared with males (ICCs r = 0.90–0.95), for sensation quality (NWC: ICC males r = 0.66, female r = 0.95; PRI ICC males r = 0.70, females r = 0.93) and slightly higher reliability for pain rating (ICCs males r = 0.90–0.95; females r = 0.92–0.96) (Table 3). Between-subjects variance also tended to show a gender differences, with females exhibiting lower group variance than males for both CPT values and pain ratings at CPT (Tables 4 and 5): mean group CV% for CPT across all test sessions, females 64.5%, males 79.2%; mean group CV% for pain rating across all sessions, females 73.3%, males 81.0%.

**Table 4:** Between-subject variance for cold response measures, females only (n = 25).

		Test Session 1				Test Session 2				Test Session 3			
	Site	Mean	SD	IQ Range	Group CV%	Mean	SD	IQ Range	Group CV%	Mean	SD	IQ Range	Group CV%
**CPT**	Thenar	12.72	7.27	9.78	**57.1**	13.15	6.64	11.15	**50.5**	12.68	6.46	10.44	**51.00**
**(°C)**	Forearm	13.99	9.47	16.09	**67.7**	13.11	8.55	15.5	**65.2**	13.45	7.78	12.18	**57.9**
	Tib Ant	11.06	9.10	20.67	**91.3**	11.70	9.68	17.75	**82.8**	10.30	8.62	15.36	**83.7**
	Foot	12.12	7.04	9.66	**58.2**	10.83	5.87	6.24	**54.3**	10.24	5.55	7.50	**54.17**
**VAS**	Thenar	3.73	2.46	4.25	**66.0**	3.55	2.47	3.75	**69.4**	4.13	2.80	3.75	**67.9**
**(/10)**	Forearm	3.83	2.46	4.25	**64.4**	3.68	2.62	3.25	**71.4**	3.65	2.73	3.50	**74.9**
	Tib Ant	3.00	2.54	2.00	**84.6**	3.30	2.93	2.00	**88.8**	3.13	2.72	2.00	**87.0**
	Foot	3.63	2.26	3.00	**62.3**	3.43	2.23	2.5	**65.2**	3.43	2.64	3.50	**77.1**

CPT Cold Pain Threshold; VAS Visual Analogue Scale; NWC Number of Words Chosen; PRI Pain Rating Index

**Table 5:** Between-subject variance for cold response measures males only (n = 20).

		Test Session 1				Test Session 2				Test Session 3			
	Site	Mean	SD	IQ Range	Group CV%	Mean	SD	IQ Range	Group CV%	Mean	SD	IQ Range	Group CV%
**CPT**	Thenar	8.36	5.63	8.04	**67.3**	9.77	6.74	10.67	**69.1**	8.45	6.04	11.69	**71.5**
**(°C)**	Forearm	8.40	7.56	10.11	**90.0**	9.11	7.37	11.86	**80.9**	8.50	6.24	9.25	**73.4**
	Tib Ant	7.13	7.43	14.06	**104.2**	5.96	6.15	10.52	**103.2**	5.70	5.42	9.18	**95.1**
	Foot	7.44	5.33	8.48	**71.7**	6.70	4.05	5.2	**60.4**	6.08	3.84	6.18	**63.2**
**VAS**	Thenar	3.28	2.41	4.25	**73.7**	3.23	2.38	5.13	**73.7**	3.03	2.45	5.25	**80.9**
**(/10)**	Forearm	3.43	2.67	3.88	**78.0**	3.13	2.33	3.88	**74.5**	2.75	2.38	5.00	**86.4**
	Tib Ant	2.15	1.60	4.75	**74.4**	1.75	1.43	5.75	**81.6**	1.95	1.81	5.25	**92.6**
	Foot	2.75	2.00	3.75	**72.6**	2.23	2.11	3.50	**94.9**	2.35	2.08	4.38	**88.4**

CPT Cold Pain Threshold; VAS Visual Analogue Scale; NWC Number of Words Chosen; PRI Pain Rating Index

## Discussion

This study evaluated the within-subject reliability and between-subject variance of response to cold over three days in healthy individuals.

### Cold pain threshold

Within-subject reliability for CPT across three sessions was excellent, with ICCs between r = 0.93 and r = 0.94 and between-day CV% less than 10% for all sites (Table 1). Comparable test-retest studies using ICCs report similarly high CPT coefficients: from r = 0.87 to r = 0.94 for finger, hand and foot values [19, 20, 30]. Cumulatively, these results suggest that, for an individual, CPT is a reliable measure over time. In contrast, between-subjects variation in CPT was wide. Group CV% on Day 1 ranged from 62% at the hand to nearly 92% at tibialis anterior (Table 2). This distinction between high individual reliability yet wide variance between individuals is supported by previous studies [19, 20, 30]. It is this considerable population variability that has resulted in the view that CPT is an unusable QST measure [16,19]. Yet CPT consistently shows high individual consistency for repeated measurement at a specific site.

### Pain intensity and sensation quality at CPT

A similar pattern of individual consistency but population variance was shown for both VAS pain intensity and sensation quality (NWC, PRI) at CPT (Tables 1 and 2). There are very few studies with which to compare these data. For VAS rating, Wasner and Brock [20] used numeric rating scales for pain at CPT at the hand, reporting similar means and a similarly high correlation between ratings of r = 0.90 (p < .001) over 24 hours. For between-subjects variability, Kelly at al. [31] reported within-subjects standard deviation of 1.4/10 for VAS pain at CPT, yet a large between-subjects standard deviation of 5.7/10, also supporting the current study. Very few studies have assessed quality of sensation at CPT and none have reported it in sufficient depth for meaningful comparison. The current study is therefore the first to investigate comprehensively sensation quality at CPT for healthy individuals. Clearly more data is needed, but this study suggests that both pain rating and sensation quality at CPT are individually determined and reliable over time. However, as for CPT, there is considerable variation in these ratings between individuals. Indeed a ‘normal’ response for some may even include paradoxical or noxious qualities normally assumed to reflect pathology. The nature of this variability in pain-free individuals warrants further clarification and investigation.

### Influence of test site

Whilst reliability at each site was high, there was a clear difference between sites for CPT, with values consistently higher at upper limb compared with lower limb sites (mean 11.2°C versus 9.1°C). This site difference is consistent with many other studies [19, 32, 33]. Cold detection in the current study was also significantly less sensitive at the foot and this is supported by the only other comparable study [20]. Strong within-subject reliability for each site appears to indicate the robustness of measures of cold pain and detection.

### Influence of gender

This study found that healthy females reported CPT at higher temperatures, although the actual sensory experience does not appear to differ, with no gender difference in pain rating or sensation quality at CPT. Many factors have been suggested to explain the influence of gender, such as differences in central pain modulation [34] or different societal mores [35]. Previous data regarding the effect of gender on CPT is extensive but inconsistent [36], with some supporting a significant gender difference [37] and some disagreeing [31,20]. The current study also found that females were more reliable in all cold response measures. Wasner and Brock [20] also reported that females were more reliable than males for CPT, even over 21 days.

### Study limitations

A number of limitations must be acknowledged. Although only Caucasian participants were included in order to limit ethnicity as a confounder, the study needs to be validated with different ethnic samples. Due to testing time restrictions, McGill descriptors were only assessed once on each test occasion at the end of CPT testing. It is therefore still unclear whether cold pain sensation quality is a reflection of central sensory interpretation and so consistent across body regions. This should be further investigated as exploration of widespread CPT sensation quality may be important in understanding the mechanisms involved in central pain processing.

## Conclusion

Cold pain testing has been discounted by some as of limited value due to poor reliability [16,19,31,32]. However this study found that, although population variance was high, individual subject reliability for all measures of cold response was excellent. The addition of pain intensity and sensation quality measures at threshold provides a more comprehensive characterization of response and may assist future interpretation of the mechanisms influencing normal and abnormal pain processing.
